# Supplementary material for: Forming global estimates of self-performance from local confidence
Source: Nat Commun. 2019 Mar 8;10:1141. doi: 10.1038/s41467-019-09075-3 (PMC6408496; doi:10.1038/s41467-019-09075-3)
Supplement: Supplementary file 1 — Supplementary Information [file 41467_2019_9075_MOESM1_ESM.pdf]

Supplementary Information

Forming global estimates of self-performance from local confidence

Rouault et al.

*Correspondence: marion.rouault@gmail.com

Supplementary Methods

Hierarchical learning model

Although learning about self-performance in the absence of feedback remains poorly characterised, we are able to draw upon extensive formal frameworks characterizing learning in the presence of external feedback. Here, we developed a candidate hierarchical learning model to characterise the process of constructing global self-performance estimates (SPEs) over the course of a variable-duration block (as used in Experiments 2 and 3). The model was composed of two hierarchical levels: 1) a perceptual module which generates a perceptual choice and confidence on each trial, and 2) a learning module which updates global SPEs across trials from local decision confidence and feedback, and is then used to make task choices at the end of blocks.

The perceptual module was grounded in signal detection theory (SDT) and generates a perceptual choice and confidence estimate on each trial. Sensory evidence X is drawn from one of two Gaussians depending on whether the greatest dot number is on the left or right of the screen ($d \in [-1\ 1]$) (Supplementary Fig. 4a). The distance between the Gaussian means (equivalent to d' in SDT) is controlled by the dot difference δ and a subject-specific sensitivity parameter k:

$$X \sim N(dk\delta, 1)$$

(Equation 1)

Under flat priors (no prior preference towards left or right), perceptual choices are made by comparing the sample X to 0 (X>0 indicating a choice of the righthand box). Confidence is then computed as a posterior probability of having chosen the correct action given the evidence (dot difference):

$$P(d = choice|X, choice):$$

$$confidence = P(d = 1|X, choice)\ if\ choice = 1,$$

$$1 - P(d = 1|X, choice)\ if\ choice = -1$$

(Equation 2)

Using Bayes rule and assuming flat priors, the relevant posterior over d can be obtained as:

$$P(d|X) = \frac{P(X|d)}{\Sigma d \; P(X|d)}$$

(Equation 3)

We introduced two different sensitivity parameters, one for perceptual choice k_{ch} and the other for confidence k_{conf}, thereby allowing the possibility that subjects have a differential sensitivity at the confidence-rating stage. For instance, subjects may continue to accumulate evidence after they made a choice, leading to a 'better' use of evidence for confidence than for choice, or additional noise may corrupt a metacognitive representation of performance, leading to 'poorer' use of evidence for confidence than for choice [1].

To determine k_{ch}, we computed each participant's psychophysical capacity d':

$$d' = \phi^{-1}\text{ (performance)} - \phi^{-1}\text{ (1-performance)}$$

(Equation 4)

where ϕ^{-1} denotes the inverse of the normal cumulative distribution function. Assuming a neutral criterion, we could then extract the mean of each Gaussian: ± $d'/2$, hence the sensitivity k_{ch} for each participant is equal to $k_{ch} = \frac{d'}{2\delta}$ with the dot difference δ reflecting the average dot difference across easy and difficult conditions. We note that for the range of d' values we observed in our participants, the distributions of internal evidence generated from easy and difficult stimuli are expected to overlap considerably. This precludes straightforward inference about the difficulty of individual stimuli.

In the learning module, we modeled self-performance estimates (SPE) as beta distributions over expected performance for each of both tasks:

$$\text{SPE} \sim B(\alpha, \beta)$$

(Equation 5)

Over the course of a block, the parameters of the beta distributions were updated as follows:

$$\begin{cases} \alpha = \alpha + 1 \; for\ correct\ trials \\ \beta = \beta + 1 \; for\ incorrect\ trials \end{cases} \text{in the presence of feedback}$$

$$\begin{cases} \alpha = \alpha + confidence \\ \beta = \beta + (1 - confidence) \end{cases} \text{in the absence of feedback}$$

(Equation 6)

Over the course of trials, the distribution mean $\frac{\alpha}{\alpha+\beta}$ naturally converges towards the true expected performance (Supplementary Fig. 4b). An interesting property of this model is that over the course of trials, distributions become narrower around expected performance slightly more rapidly with feedback than without (Supplementary Fig. 4b). This is due to the confidence update being shared between α and β parameters in the absence of feedback (reflecting uncertainty about the true performance), whereas only one of the two parameters is updated in the presence of feedback. At the end of the block, one of the two tasks is then chosen via comparing the posterior distributions of SPEs across both tasks. The model has one free parameter, k_{conf}.

At the start of each learning block, SPEs for both tasks were initialised at an expected performance of 66% correct (α=6 and β=3). This prior embodies the assumption that average performance expectations are neither at ceiling nor at floor. We additionally checked that other prior values (e.g. α=1, β=1) did not strongly affect task choices – for all but the shortest block durations, posterior distributions converged rapidly on expected accuracy regardless of the choice of initial α and β values.

We performed model fitting and simulations of this hierarchical learning model. The single free parameter k_{conf} was fitted for each subject by maximising the log-likelihood of task choices observed in Experiment 2. Note that this is a relatively simple optimisation problem over a single free parameter. Specifically, 5000 possible k_{conf} values were randomly sampled from a uniform distribution between upper and lower bounds set at 0.028 and 0.0001 respectively, which corresponds to a range between 2.35 and 0.008 in d' units, and log-likelihood was calculated for each k_{conf} value. The model's perceptual choices were conditioned on subjects' actual choices during parameter fitting. To obtain predictions for task choices, we then performed 200 simulations using each subjects' k_{ch} and best-fitting k_{conf} (Supplementary Fig. 4c). One subject was removed here for an unreliable fit. In these simulations, the model made both perceptual choices on each trial and task choices at the end of block;

the final distributions used for predicting task choices in each simulation were averages over 1000 iterations of each block to accommodate variability in sequences of perceptual choice.

Generalisation of hierarchical learning model to Experiment 3

In Experiment 3, instead of getting no feedback, participants were asked to report their confidence in their performance on a trial-by-trial basis. We were able to use this direct confidence report in place of the confidence derived from the perceptual module in simulations for Experiment 3, removing the need for fitting a sensitivity parameter. As for Experiment 2, we performed simulations of the learning module using participants' actual trial sequences, including perceptual choices, feedback and confidence data, and plotted the obtained simulations (n=200) on top of participants' learning curves for the six types of block (Supplementary Fig. 4e and Supplementary Notes).

Alternative accounts

While the proposed model constitutes a possible formalization of how SPEs are built, the current experimental protocol was not designed to discriminate amongst competing models. Due to the blocked structure of our experiments, only one task choice per condition per subject was available in Experiments 2 and 3 (and two per subject in Experiment 1), leading to limited data points for model fitting and parameter recovery [2]. This block structure was necessary to probe aggregation of confidence over time, and to examine whether subjects use variability in local confidence across blocks when forming global SPEs. Our simulations instead provide a proof of principle that a Bayesian learning scheme similar to the one suggested here could account for the formation of SPEs (Supplementary Fig. 4 and Supplementary Notes).

Supplementary Notes

In Experiment 1, we examined whether variability in objective performance affected SPEs. In a linear regression on subjective task ratings we obtained a main effect of objective performance (mean β=.40, $p<10^{-11}$) and a main effect of feedback presence (mean β=.42, $p<10^{-8}$) together with a significant interaction (mean β=.23, $p<10^{-10}$), again due to SPEs increasing in the presence of feedback, an increase which was most prominent for tasks in which subjects performed better.

Critically, variations in global self-performance estimates (SPEs) could not be trivially explained by differential fluctuations in objective performance over time in a subset of blocks. First, performance did not improve or degrade between the first and second halves of the experiment ($|t_{28}|<.57$, $p>.58$; except for an improvement on Easy-No-Feedback tasks: $t_{28}=-2.78$, $p=.01$). We found a small but significant decrease in overall RTs (all $t_{28}>2.30$, all $p<.03$), but importantly RTs did not decrease more rapidly in any of the four experimental conditions (effect sizes: all $t_{28}<0.64$, all $p>.53$).

In addition, we investigated whether subjects considered the whole learning block when forming end-of-block SPEs or whether they gave more weight to information from the most recent trials (see Methods). We found that the recent difference in accuracy (last quartile) strongly influenced task choices ($t_{28}=3.10$, $p=.002$), but there was no monotonic trend with more recent trials consistently influencing more task choices (Supplementary Fig. 1).

In Experiment 2, replicating Experiment 1, subjects chose easy tasks more often than difficult ones, again indicating that SPEs were related to fluctuations in difficulty level (Supplementary Fig. 2b). For instance, subjects were equally likely to choose a Feedback-Difficult task when paired with a No-Feedback-Easy task, despite performing significantly better at the latter (Fig. 4b).

In Experiment 3, we sought further support for our proposed hierarchical learning model in explaining global SPEs formation on an independent data set. In Experiment 3, instead estimates of trial-by-trial confidence obtained from the model's perceptual module, we directly entered subjects' confidence ratings into simulations of the learning module. In keeping with our results for Experiment 2, the model was (1) able to capture qualitative features of subjects' SPEs across most of the block types (Supplementary Fig. 4d) and (2) tasks with external feedback were chosen more frequently by the model than by subjects (Supplementary Fig. 4d, lower-left panels), indicating that more work is required to understand the origin of this stronger inclination for tasks with external feedback.

To further assess the reliability of the MLE estimates of meta-*d'*, we performed additional parameter recovery simulations. Specifically, we generated confidence rating data from N=46 simulated subjects with 90 trials per subject following the procedures outlined in [3]. The group metacognitive efficiency was set to 0.8, and individual subject meta-*d'*/*d'* values were sampled from a Gaussian distribution centered on *d'*=1.55 (the mean *d'* value we observed for Experiment 3 data across easy and difficult conditions) with SD=0.5. We sampled confidence rating counts for known meta-*d'*/*d'* values using the metad_sim function from the HMeta-d toolbox (https://github.com/metacoglab/HMeta-d), keeping confidence rating criteria fixed across subjects. We observed that the ground truth meta-*d'* values were recovered much more accurately when using the hierarchical compared to the MLE fits (Supplementary Fig. 5d).

Supplementary Figures

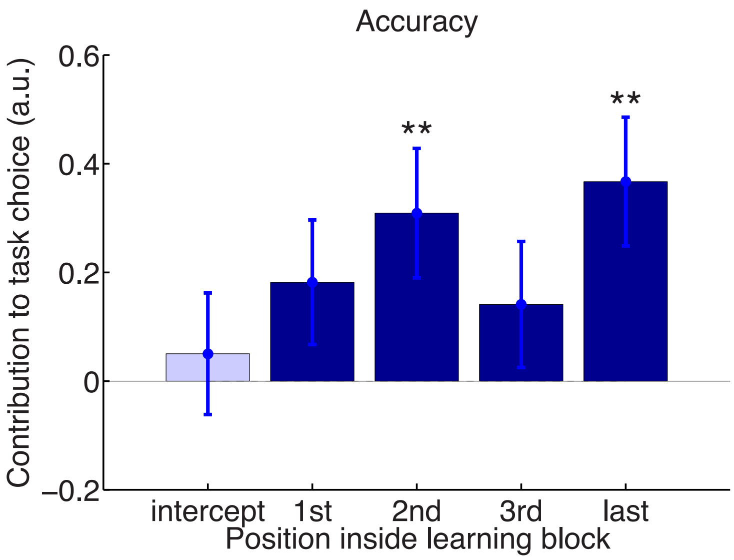

Supplementary Figure 1. Order effects in Experiment 1 (N=29). Stars indicate a significant effect of the difference in accuracy between tasks on task choices as assessed by a logistic regression (*p**<.005, statistical significance of the regression coefficients). The four bars correspond to the four quartiles of learning blocks (see Methods). Error bars are SE.**

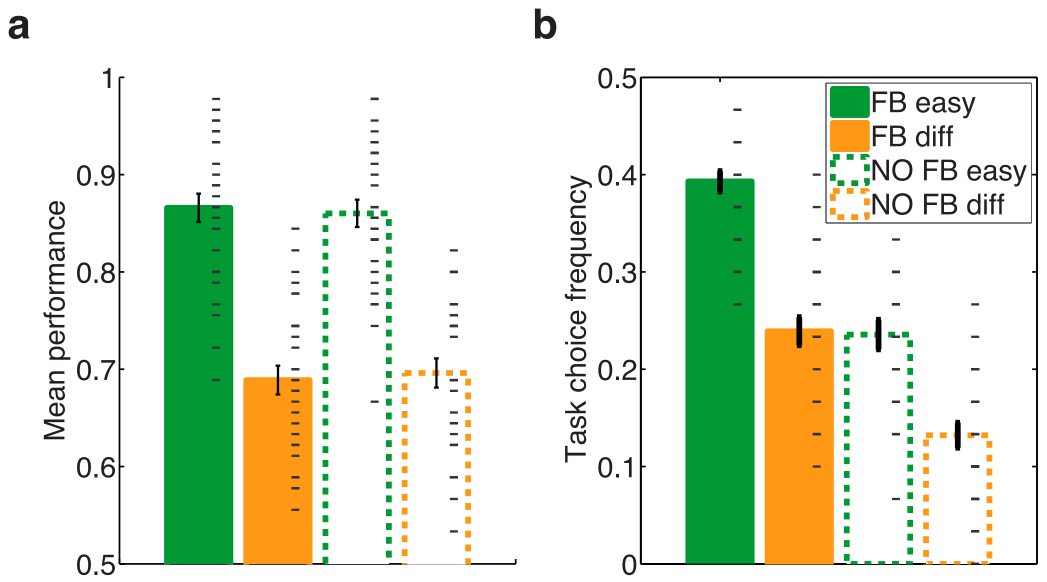

Supplementary Figure 2. Behavioral dissociation between objective and perceived performance in Experiment 2 (N=29) replicating the findings of Experiment 1. a, Performance (mean percent correct) was better for easy (green) than difficult (orange) tasks, but was not different in tasks with (plain lines) and without (dotted lines) feedback. b, Self-performance estimates were higher in the presence of feedback, despite objective performance being unaffected. Error bars represent S.E.M across subjects. Black dashes are individual data points (note that there are fewer performance data points visible than N=29 as several subjects had the same performance, due to blocks having fewer trials than in Experiment 1). See also Fig. 4.

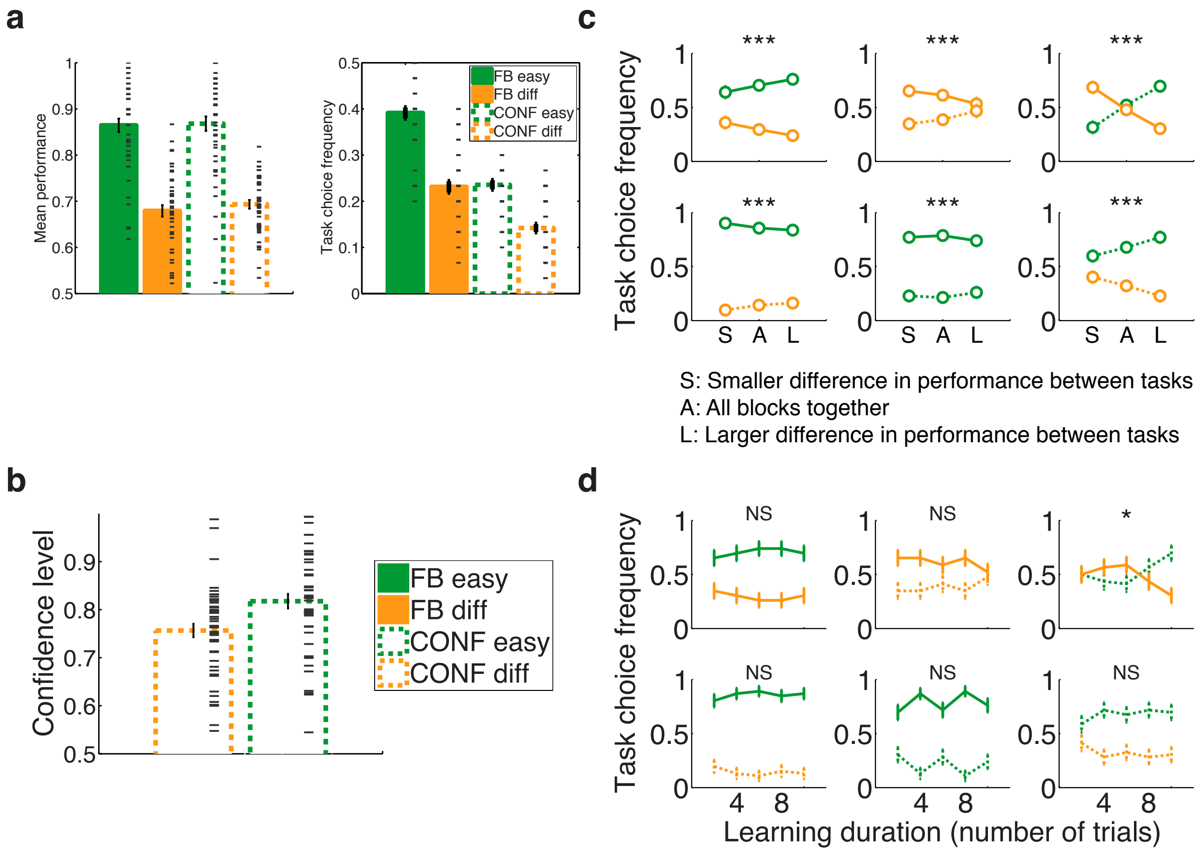

Supplementary Figure 3. Subjects' behavior in Experiment 3 (N=46) replicating Experiments 1 and 2

a, Behavioral dissociation between objective performance and subjective self-performance estimates. b, Confidence level (mean confidence rating) was higher for easy than difficult tasks. ****p*<.001. c, Fluctuations in local performance influenced task choice. The central circle of each subplot represents the average task choice frequency over all learning blocks ['A']. The leftmost and rightmost circles display the same data split into learning blocks with smaller ['S'] and larger ['L'] differences in objective performance between tasks. Logistic regressions confirmed a significant influence of the difference in performance between tasks on task choices with all β>3.99, all ****p*<.005. d, Task choice frequency as a function of block duration in the six task pairings. Subjects traded-off getting explicit feedback against their subjective self-performance estimates. **p*=.03 indicates significance of logistic regression coefficient; NS indicates that block duration had no significant influence on task choice (see Methods). Error bars indicate S.E.M. across subjects. Black dashes are individual data points (few task choice data points due to a limited number of blocks per subject). See also Fig. 5.

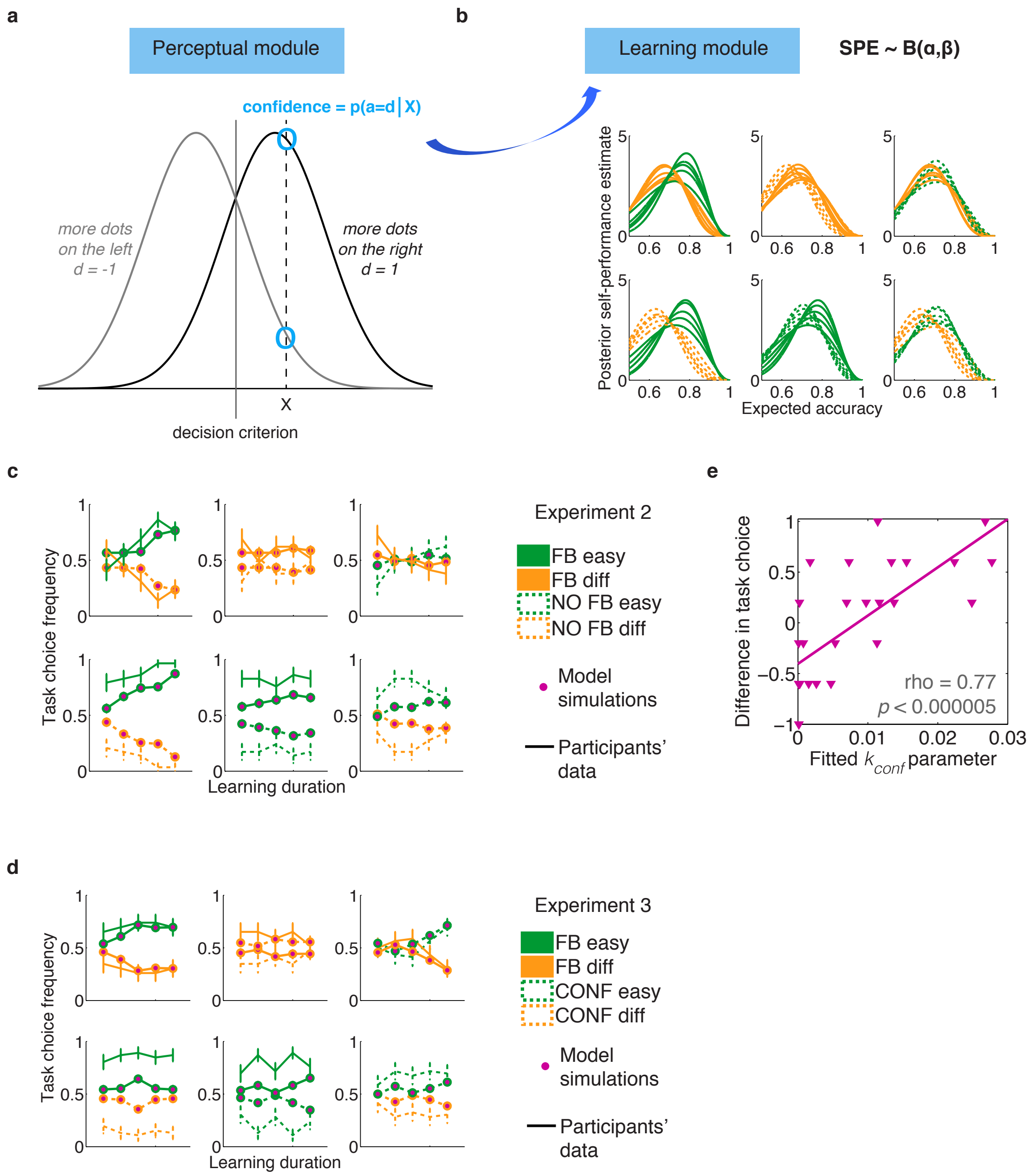

Supplementary Figure 4. Hierarchical learning model

a, A perceptual module grounded in signal detection theory predicted perceptual choice and associated (local) confidence on each trial. *d* is the true state of the world, *a* the selected action and *X* the evidence sampled on a given trial. Confidence corresponds to the probability that the perceptual choice was correct given *X*. b, Learning module. Self-performance estimates (SPEs) were modeled as beta distributions over expected performance and parameters were updated using feedback when available or, in the absence of feedback, confidence from the perceptual module. Task choices were made by comparing posteriors over SPEs at the end of blocks. Posteriors over SPEs at the end of blocks are displayed for an example subject for the five possible block durations and the six possible types of block. SPEs converge towards the average expected accuracy over the course of learning. See Supplementary Methods for details. c-d, Hierarchical learning model simulations (n=200, pink circles) plotted together with subjects' task choice frequencies (curves) as a function of block duration for all six block types in Experiment 2 (c) and Experiment 3 (d). Error bars represent S.E.M over simulations and participants respectively. e, The difference in frequency of selecting the Easy-No-Feedback task over the Difficult-Feedback task (third panel in Supplementary Fig. 4c) correlated with k_{conf} parameter for each subject in Experiment 2. See also Results and Supplementary Notes.

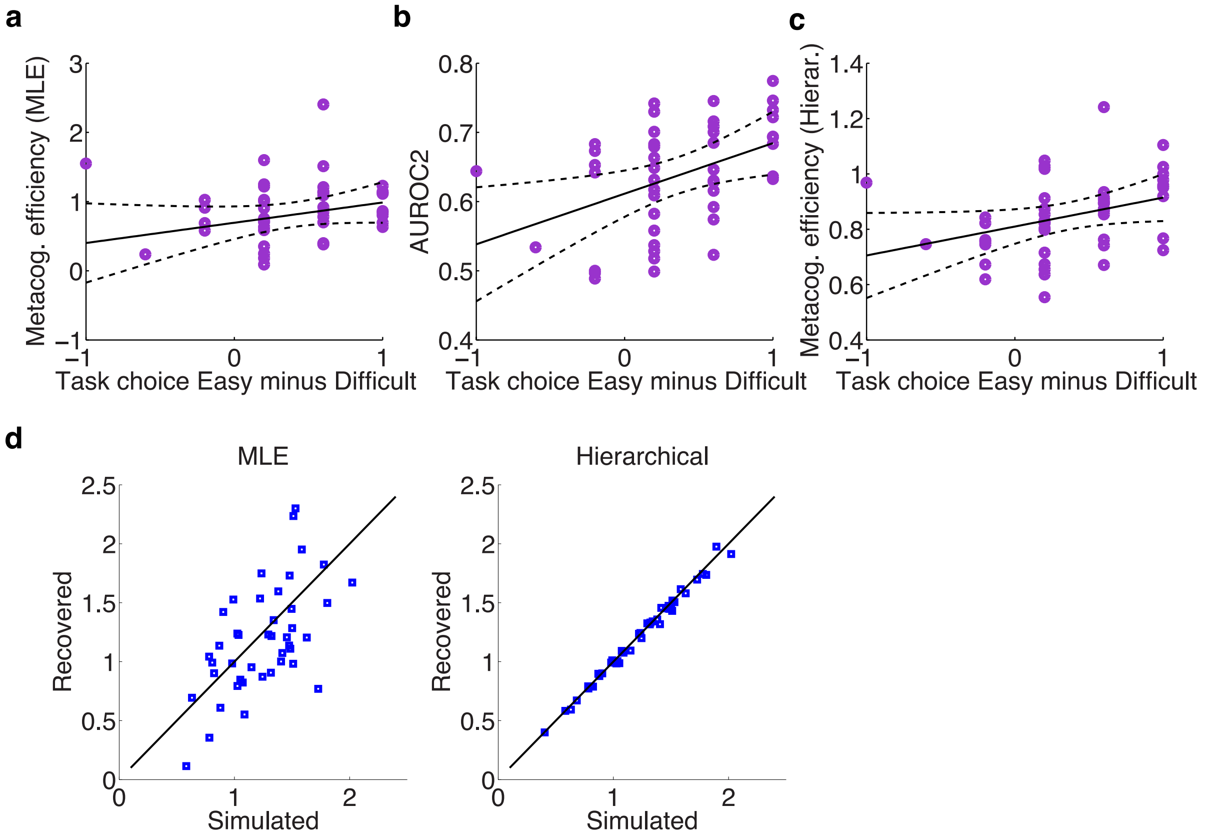

Supplementary Figure 5. Relationship between three measures of metacognitive ability and global SPEs

Between-subjects correlations between metacognitive ability and task choices. Purple dots are subjects' data (N=46), dotted lines are 95% confidence intervals. Both metacognitive efficiency (meta-*d'*/*d'*) when estimated hierarchically (c, identical to Fig. 5d) (Pearson ρ=.35, *p*=.02, Spearman ρ=.43, *p*=.003) and metacognitive ability estimated as the area under the type 2 receiver operating curve (AUROC2) (b) (Pearson ρ=.44, *p*=.0024, Spearman ρ=.45, *p*=.0016) showed that subjects with better metacognition were also better at selecting the easiest of both tasks in end-of-block task choices, whereas there was no significant association for metacognitive efficiency as estimated using a maximum likelihood fit (MLE) (a) (Pearson ρ =.12, *p*=.45, Spearman ρ=.21, *p*=.18). d. Parameter recovery indicating that meta-*d'* estimation was more reliable when estimated hierarchically as compared to MLE (see Supplementary Methods). This difference in reliability indicates more credence should be given to the correlation identified via the hierarchical fit in panel c.

Supplementary References

1. Fleming, S. M. & Daw, N. D. Self-evaluation of decision-making: A general Bayesian framework for metacognitive computation. *Psychological Review* **124,** 91–114 (2017).
2. Palminteri, S., Wyart, V. & Koechlin, E. The Importance of Falsification in Computational Cognitive Modeling. *Trends in Cognitive Sciences* (2017).
3. Fleming, S. M. HMeta-d: hierarchical Bayesian estimation of metacognitive efficiency from confidence ratings. *Neurosci Conscious* **2017,** 1–14 (2017).
